# Self-Guided Mental Health Apps Targeting Racial and Ethnic Minority Groups: Scoping Review

**DOI:** 10.2196/48991

**Published:** 2023-12-06

**Authors:** Fiby Saad, Mia Eisenstadt, Shaun Liverpool, Courtney Carlsson, Isabella Vainieri

**Affiliations:** 1 Clinical, Educational and Health Psychology, Division of Psychology and Language Sciences University College London Faculty of Brain Sciences London United Kingdom; 2 Evidence Based Practice Unit Anna Freud National Centre for Children and Families London United Kingdom; 3 Department of Social Work & Wellbeing Edge Hill University Faculty of Health, Social Care and Medicine Ormskirk United Kingdom; 4 Paradym London United Kingdom; 5 Department of Psychology Royal Holloway, University of London Egham, Surrey United Kingdom

**Keywords:** mental health apps, racial and ethnic minority groups, self-guided, mental health, culturally appropriate technology

## Abstract

**Background:**

The use of mental health apps (MHAs) is increasing rapidly. However, little is known about the use of MHAs by racial and ethnic minority groups.

**Objective:**

In this review, we aimed to examine the acceptability and effectiveness of MHAs among racial and ethnic minority groups, describe the purposes of using MHAs, identify the barriers to MHA use in racial and ethnic minority groups, and identify the gaps in the literature.

**Methods:**

A systematic search was conducted on August 25, 2023, using Web of Science, Embase, PsycINFO, PsycArticles, PsycExtra, and MEDLINE. Articles were quality appraised using the Mixed Methods Appraisal Tool, and data were extracted and summarized to form a narrative synthesis.

**Results:**

A total of 15 studies met the inclusion criteria. Studies were primarily conducted in the United States, and the MHAs designed for racial and ethnic minority groups included ¡Aptívate!, iBobbly, AIMhi- Y, BRAVE, Build Your Own Theme Song, Mindful You, Sanadak, and 12 more MHAs used in 1 study. The MHAs were predominantly informed by cognitive behavioral therapy and focused on reducing depressive symptoms. MHAs were considered acceptable for racial and ethnic minority groups; however, engagement rates dropped over time. Only 2 studies quantitatively reported the effectiveness of MHAs among racial and ethnic minority groups. Barriers to use included the repetitiveness of the MHAs, stigma, lack of personalization, and technical issues.

**Conclusions:**

Considering the growing interest in MHAs, the available evidence for MHAs for racial and ethnic minority groups appears limited. Although the acceptability seems consistent, more research is needed to support the effectiveness of MHAs. Future research should also prioritize studies to explore the specific needs of racial and ethnic minority groups if MHAs are to be successfully adopted.

## Introduction

### Background

Mental health apps (MHAs) are frequently used as self-guided tools to help people with various mental health conditions, including anxiety [1] and depression [2]. More than 10,000 MHAs are currently available for smartphone users [3], and this number is increasing daily [4] due to a high interest in MHAs among the public [5], which peaked during the COVID-19 pandemic [6]. For instance, the number of MHA downloads increased by 2 million during the COVID-19 pandemic compared with prepandemic levels [6].

Despite the overall increase in downloads over the years, MHAs appear to appeal to certain populations more than others. For instance, people who have had a previous diagnosis of mental illness [7] or those who are more symptomatic [8] may be more likely to download MHAs. Interest in MHAs is especially high among younger generations, with studies reporting that younger participants (aged 18-22 years) were more interested in MHAs than older participants (≥23 years) [7,9]. This increased interest in MHAs among younger generations could be attributed to the incorporation of smartphone technology in their daily lives [5], as well as the increase in mental health conditions among young people [10]. Another reason is self-monitoring and tracking of progress over time, as it can influence an individual’s motivation to continue psychological treatment and enhance feelings of control, which is especially important in young people [11].

The COVID-19 pandemic has significantly impacted the mental health of young people. For example, the Opinions and Lifestyle Survey conducted by the Office for National Statistics revealed that the prevalence of anxiety and depression increased by almost 11% between June 2019 and March 2020 in people aged 16 to 39 years compared with prepandemic levels. However, studies have shown that 50% to 80% of young adults who struggle with mental health issues do not seek treatment [12,13] Some experts argue that stigma around mental illness is a key barrier when accessing face-to-face (FTF) therapy, leaving some young people to express a preference for MHAs [14,15]. Overall, younger age and high self-stigma are associated with a low mental health help–seeking attitude and a negative attitude toward FTF therapy [16].

Despite their popularity, MHAs present some challenges for app users. First, there seems to be a high turnover rate of MHAs. Larsen et al [17] found that apps targeted for depression were unavailable to access approximately every 3 days, leading to difficulties for users to commit to one app and see any long-term benefits. Another major issue with MHAs is the level of user engagement because people rarely use MHAs as a long-term solution [18,19]. For instance, studies have shown that the median duration of app use was only 3 hours over an 8-week treatment period [19] and the median retention rate was 5.5 days (across 8 studies) [20].

However, the most important issue with MHAs is the lack of evidence of their effectiveness. A recent review showed that only 2 out of the 73 apps targeting common mental health symptoms provided direct evidence to support the use and effectiveness of their app [17]; this highlights that app developers might use scientific jargons to lure users into using the app despite no evidence supporting their claims. Even apps that are approved by public authorities report little evidence of their effectiveness. Another review found that only 15% of the MHAs in the UK National Health Service library provided evidence of effectiveness [21], highlighting the need for regulations to ensure that MHAs meet specific standards of care [22].

Despite these challenges, there are a range of benefits that have contributed to the rapid growth and popularity of MHAs. First, MHAs can be accessed anywhere and at any given time. By contrast, traditional therapy occurs at set hours or in specific settings. Furthermore, services may have increased waiting times [23], which raises major risks for individuals, such as self-harm or suicide [24]. Second, unlike FTF therapy, MHAs can be used by any number of people. Third, unlike publicly funded therapy, in which an individual requires a diagnosis or a basis for referral, MHAs generally have no requirements or criteria for use. Overall, MHAs can be used outside clinical settings or as adjunct support to help people manage everyday stress [25].

The ability to access mental health aid outside clinical settings can be especially helpful for people from racial and ethnic minority backgrounds. For this review, *racial and ethnic minority group* refers to any racial and ethnic group with national or cultural traditions different from those of the main majority. Evidence shows that people from racial and ethnic minority backgrounds experience higher levels of stressors than the majority population; these stressors such as low socioeconomic status, discrimination, and racism can negatively affect mental health outcomes [26-28]. People from racial and ethnic minority backgrounds also experience increased barriers when engaging with mental health services [29-31] and are less likely to self-report and receive treatment [31]. This is possibly due to personal and environmental barriers such as the inability to recognize and accept mental health problems, embarrassment, confidentiality concerns, preference for self-reliance, social stigma against mental health, and financial factors [32-36]. Other factors are related to health care providers, such as language barriers, cultural naivety, insensitivity, and discrimination toward the needs of racial and ethnic minority service users [36]. Overall, individuals from racial and ethnic minority backgrounds are exposed to increased risk factors for poor mental health and experience inequalities in accessing mental health care.

MHAs can offer opportunities to access mental health support and overcome some of the abovementioned barriers encountered by racial and ethnic minority populations. For instance, MHAs provide a sense of safety to some users, increasing their ability to disclose and share their feelings [37], as they enable access to services from their homes, and more importantly, they avoid the stigma associated with disclosing a mental health problem [38,39]. This is particularly important for racial and ethnic minority populations, as evidence suggests that mental health stigma is higher in people from racial and ethnic minority backgrounds than in the majority population [33]. Furthermore, the consequences of mental health stigma are higher among racial and ethnic minority populations, as they often experience other social adversities that negatively affect mental health, leading to untreated mental health problems as well as poorer mental health outcomes [33]. Kern et al [7] conducted a survey of college students in the United States to explore their openness, use, and attitudes toward MHAs. Out of 565 respondents, 179 were of racial and ethnic minority background, and they found that participants from this background preferred downloading an MHA instead of going to therapy. Similarly, Lungu and Sun [40] found that Asian American youth endorsed seeking help on the web rather than going to professionals in an FTF setting. Although this is promising, interest does not always correlate with actual use [41]. Furthermore, a recent systematic review of MHAs found that there was an absence of diverse samples, with many studies using majority White populations, whereas the effectiveness, acceptability, and use of MHAs in racial and ethnic minority groups remain poorly understood [42].

### Objectives

We conducted a scoping review of the literature to (1) describe the purposes of using MHAs in racial and ethnic minority groups, (2) examine the acceptability of MHAs among those groups, (3) examine the effectiveness of MHAs with these groups, (4) identify the barriers to MHA use within these groups, and (5) identify the gaps in the literature. We will only focus on self-guided MHAs that users can use without additional help (eg, video chat and text messaging), as they offer a more sheltered environment for the user, further removing the issue of stigma [43]. Due to the recent interest in MHAs among young people and the need for a comprehensive overview of the literature focusing on racial and ethnic minority groups, this study covered a wide age range of 14 to 36 years. This age range also captures 3 main age groups that have been found to have high smartphone use:14 to 18 [15,44,45], 18 to 21 [7,43], and 25 to 36 years [46].

## Methods

This scoping review was conducted in accordance with the Joanna Briggs Institute methodology for scoping reviews [47] and the PRISMA-ScR (Preferred Reporting Items for Systematic Reviews and Meta-Analyses extension for Scoping Reviews) guidelines [48]. Refer to Multimedia Appendix 1 [49] for the PRISMA-ScR checklist.

### Search Strategy

A systematic search was conducted in the following databases: Embase; PsycINFO; PsycArticles; PsycExtra; MEDLINE ALL, via OVID; and Web of Science. See Multimedia Appendix 2 for a complete list of search terms. The search algorithm was defined including concepts related to *mobile phone apps*, *mental health*, and *racial and ethnic minority groups*. The search was conducted on August 25, 2023, with no limit placed on the publication year.

### Eligibility Criteria

Studies were included if they fulfilled all the following criteria: (1) most participants were from a racial and ethnic minority background (ie, more than 50%); (2) the study explored “self-guided” MHAs, meaning that the participants used the apps alone without outside help; (3) participants’ age range was between 14 and 36 years; (4) the study focused on mental health issues; and (5) the study was written in English. Studies were excluded if they were solely used for adherence to medication or other lifestyle changes such as diet or exercise.

### Selection Process

The CADIMA software package (Julius Kühn-Institut) was used to facilitate the review processes, including screening and data extraction [50]. The titles and abstracts were independently screened by 2 reviewers (FS and IV), and those that met our inclusion criteria were used for full-text screening. All the full texts were screened in parallel by the same 2 reviewers. Any inconsistencies between the reviewers were discussed before reaching an agreement.

### Data Extraction and Quality Assessment

The extracted data included (1) study design (eg, qualitative, quantitative, or mixed methods); (2) participants’ demographic details (eg, age, ethnicity, and occupation); (3) geographic location; (4) the intervention used, including theoretical basis, purpose, and duration of use; (5) data regarding the acceptability of MHAs; (6) data related to the effectiveness of the intervention; and (7) any barriers to MHA use. Acceptability was defined as “a multi-faceted construct that reflects the extent to which people delivering or receiving a health care intervention consider it to be appropriate, based on anticipated or experienced cognitive and emotional responses to the intervention” [51].

Critical appraisal was conducted following the Mixed Methods Appraisal Tool checklist [52]. The Mixed Methods Appraisal Tool consists of 2 general screening questions and 5 questions for each type of study design. Each question was answered by responding “yes,” “no,” or “can’t tell” and scored 1 for “yes” and 0 for “no,” resulting in the maximum score of 7 for each study. Quality assessment was conducted independently by 2 reviewers (FS and IV). Any discrepancies were discussed, and if necessary, a third team member was consulted to reach a final decision.

### Data Analysis and Data Synthesis

First, the study and its population characteristics were charted to provide an overall description of the body of evidence. Second, a narrative synthesis, supported by thematic and content analysis as outlined by Popay et al [53], was conducted to provide an overall narrative to address the aims of the review.

## Results

**Figure 1:**
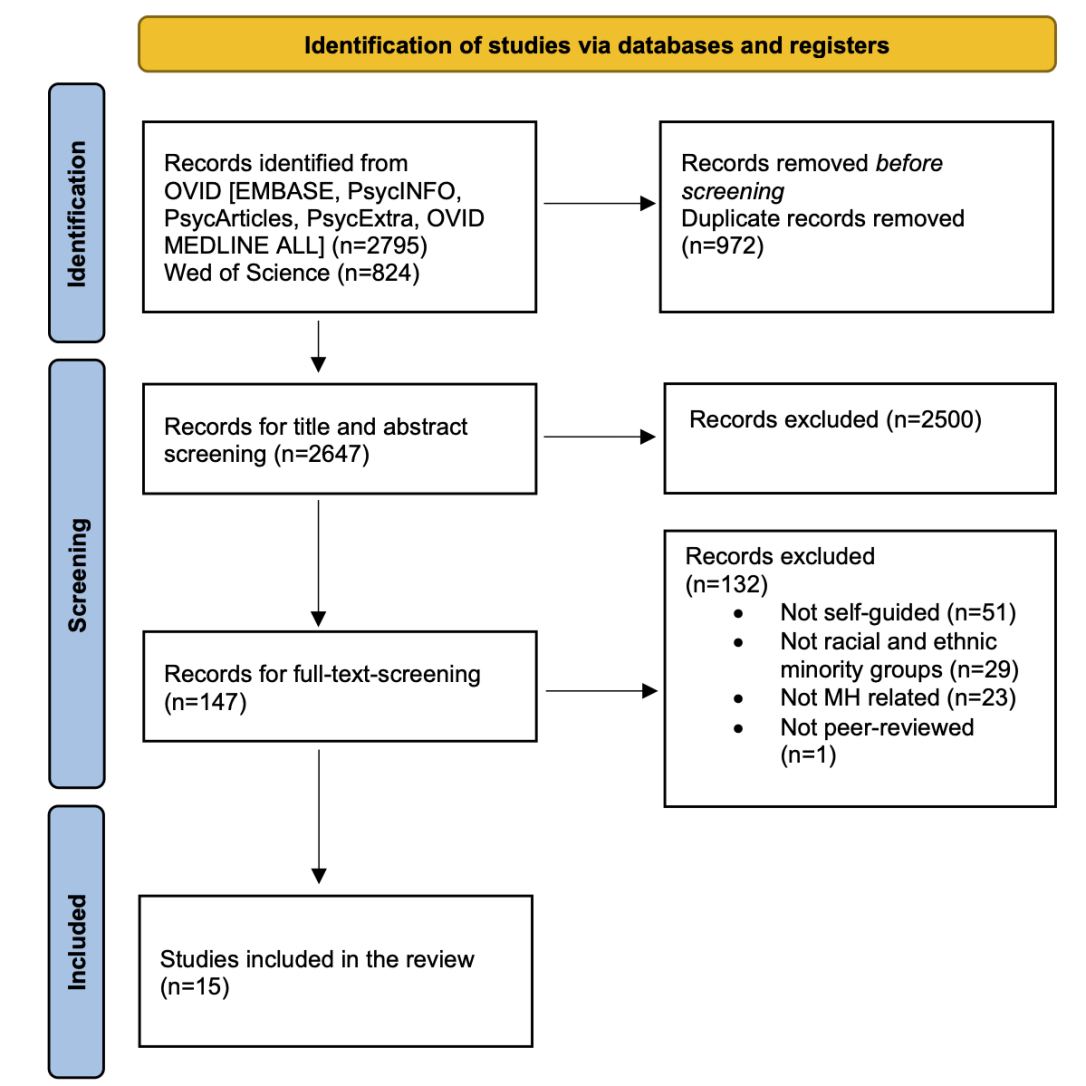
Flowchart highlighting the key stages of the screening process. MH: mental health.

A total of 15 studies were eligible for inclusion in this scoping review. A PRISMA (Preferred Reporting Items for Systematic Reviews and Meta-Analyses) flow diagram [49] is shown in Figure 1 to illustrate the flow of information and the identified records at each phase of the scoping review.

### Characteristics of Included Studies

Out of the 15 publications that met the inclusion criteria for this review [40,54-67], 10 (67%) studies were conducted in the United States [40,54,56,58-61,63,66,67], with publication dates ranging from 2016 to 2023. Overall, 13 (87%) of the 15 studies focused on specific MHAs [54-61,63-67], including 2 MHA prototypes [58,67]. Of the app-specific studies, 4 had a mixed methods design [55,58,60,63], 7 were quantitative [54,56,57,59,61,65,67] and 2 were qualitative studies [64,66]. The last 2 studies did not focus on a specific MHA; instead, they assessed preference for web-based help versus FTF mental health help using surveys, both were quantitative [40,62]. Table 1 reports the characteristics of the reviewed studies, including study name, study design, sample size, mean age of participants, the racial and ethnic minority group, MHA name (if applicable), targeted mental health condition, duration of use, outcome measure, study location, and critical appraisal score.

**Table 1:** Summary of included studies.

Study (year)	MHA^a^ name	Targeted mental health condition	Duration of use	Study design	Outcome measure	Ethnic minority group	Mean age (years)	Sample size	Geographic location	Critical appraisal score
Dahne et al [54], (2019)	¡Aptívate!	Depression	8 wk	Quantitative	Spanish language Beck Depression Inventory—II Semistructured interview	Hispanic	36.05	42	United States	6
Tighe et al [55], (2020)	iBobbly	Depression	6 wk	Mixed methods	Semistructured interview and RCT^b^ data	Aboriginal and Torres Strait Islander	24.15	Interviews (n=18) RCT (n=61)	Canada	6
Pratap et al [56], (2018)	iPST^c^	Depression	12 wk	Quantitative	PHQ-9^d^ Sheehan Disability Scale	Hispanic or Latino	34.90	Hispanic (n=106) Non-Hispanic (n=239)	United States	6
Lungu and Sun [40], (2016)	—^e^	—	—	Quantitative	18-item web-based survey MHI-21^f^	Asian American	18.7	572	United States	5
Tighe et al [57], (2017)	iBobbly	Suicidal ideation depression psychological distress impulsivity	6 wk	Quantitative	DSI-SS^g^ PHQ-9 K10^h^ BIS-11^i^	Indigenous Australians	26.25	61	Australia	6
McCall et al [58], (2021)	—	Anxiety and depression	—	Mixed methods	QUIS^j^ Tobii (eye-tracking software)	African American	29	15	United States	6
Rushing et al [59], (2021)	BRAVE	Promote help-seeking behavior, mental health, and cultural resilience	8 wk	Quantitative	None used	American Indian and Alaska Native	15-24	1030	United States	7
Stephens et al [60], (2020)	BRAVE	Promote help-seeking behavior, mental health, and cultural resilience	8 wk	Mixed methods	None used	American Indian and Alaska Native	15-24	1030	United States	
Wrobel et al [61], (2022)	BRAVE	Promote help-seeking behavior, mental health, and cultural resilience	8 wk	Quantitative	Mobile Commons tracks message engagement Questionnaires and surveys	American Indian and Alaska Native	15-24	1030	United States	7
Maloney et al [62], (2020)	—	—	—	Quantitative	Surveys	Jamaican	10-19	107	United Kingdom	6
Neal-Barnett et al [63], (2019)	BYOTS^k^	Anxiety and negative thinking	1 wk	Mixed methods	Focus groups	Black and biracial	12-15	72	United States	7
Povey et al [64], (2016)	iBobbly	Depression	1 wk	Qualitative	Focus groups	Aboriginal or Torres Strait Islander	—	—	Australia	5
Röhr et al [65], (2021)	Sanadak	PTSD^l^	4 wk	Quantitative	PDS-5^m^ PHQ-9 GAD-7^n^ PHQ-15 GSE^o^ SSMIS-SF^p^ SSMIS-AW^q^ SSMIS-AG^r^ SSMIS-AP^s^ SSMIS-HS^t^ RS-13^u^ LSNS-6^v^ ESSI^w^ EQ-5D-5L EQ-VAS^x^ PGI^y^	Syrian	Control: 33.67 Intervention: 32.98	133	Germany	6
Agapie et al [66], (2022)	Mindshift Sanvello Woebot Headspace Insight Timer Shine Smiling Mind Covid Coach Daylio Moodflow Talk Life	CBT^z^ (n=4) Mindfulness (n=4) Miscellaneous apps (n=4), which did not pertain to 1 category, including a coping app, journaling app, mood-tracking app, and peer support app	1 wk for each category	Qualitative	Follow-up survey and Mobile App Rating Scale	Hispanic and Black	18	5	United States	5
Watson-Singleton [67], (2023)	Mindful You	Mindfulness	2 wk	Quantitative	FFMQ^aa^ MSES^ab^ ATMS^ac^ MBUS^ad^ Mindfulness Knowledge Scale PSS^ae^ Difficulties in Emotion Regulation Scale	African American	31.1	39	United States	4

^a^MHA: mental health app.

^b^RCT: randomized controlled trial.

^c^iPST: internet-based problem-solving therapy.

^d^PHQ: Patient Health Questionnaire.

^e^Not available.

^f^MHI-21: Mental Health Inventory.

^g^DSI-SS: Depressive Symptom Inventory–Suicidality Subscale.

^h^K10: The Kessler Psychological Distress Scale.

^i^BIS-11: Barratt Impulsivity Scale.

^j^QUIS: Questionnaire for User Interface Satisfaction.

^k^BYOTS: Build Your Own Theme Song.

^l^PTSD: posttraumatic stress disorder.

^m^PDS: Posttraumatic Diagnostic Scale for DSM-5.

^n^GAD-7: Generalized Anxiety Disorder, 7 items.

^o^GSE: General Self-efficacy.

^p^SSMIS-SF: Self-Stigma of Mental Illness Scale–Short Form.

^q^SSMIS-AW: Self-Stigma of Mental Illness Scale– Stereotype Awareness.

^r^SSMIS-AG: Self-Stigma of Mental Illness Scale–Stereotype Agreement.

^s^SSMIS-AP: Self-Stigma of Mental Illness Scale–Stereotype Application.

^t^SSMIS-HS: Self-Stigma of Mental Illness Scale–Harm to Self-esteem.

^u^RS-13: Resilience Scale.

^v^LSNS-6: Lubben Social Network Scale (social isolation).

^w^ESSI: ENRICHD Social Support Inventory.

^x^VAS: Visual Analog Scale.

^y^PGI: Posttraumatic Growth Inventory.

^z^CBT: cognitive behavioral therapy.

^aa^FFMQ: Five Facet Mindfulness Questionnaire.

^ab^MSES: Mindfulness Self-Efficacy Scale.

^ac^ATMS: Attitudes Toward Mindfulness Scale.

^ad^MBUS: Mindfulness Behavior Usage Scale.

^ae^PSS: Perceived Stress Scale.

### Purposes of Using MHAs in Racial and Ethnic Minority Groups

The most common purpose for using MHAs was depression (¡Aptívate!, iPST [internet-based problem-solving therapy], iBobbly, and a prototype app by McCall et al [58]) [54,56-58]. The iBobbly MHA also addressed impulsivity; however, this was not the main purpose of use.

Two studies focused on overall psychological well-being [59,66]. The BRAVE app was used to promote overall mental well-being by including help-seeking behaviors, general mental health, and cultural resilience as outcome measures. Agapie et al [66] included a mix of MHAs, with the aim of measuring their effect on psychological well-being using qualitative methods.

The other apps in this review had various purposes. The Build Your Own Theme Song (BYOTS) app was aimed at reducing anxiety and negative thoughts. The Sanadak app [65] aimed to reduce posttraumatic stress disorder (PTSD) symptoms, whereas Mindful You [67] aimed to reduce stress.

### Intervention Characteristics

A total of 20 apps were investigated in this review; 8 MHAs (including 2 prototypes) were included in 14 of the 15 studies [54,56-59,63-67], whereas 1 study [66] included 12 self-help MHAs that were qualitatively investigated.

Of the 20 apps, 8 (40%) were based on cognitive behavioral therapy (CBT) [65,66] or variations of the CBT approach, such as problem-solving therapy [56], acceptance-based therapy [57], and behavioral activation therapy [54]. Five (25%) apps were based on mindfulness [66,67], and the 7 (35%) apps could be described as miscellaneous: the prototype by McCall et al [58], which included the elements of CBT and psychotherapy [58]; the BYOTS app, which is based on musical cognitive restructuring [63]; and the BRAVE app, which is based on offering information and role model videos aimed at providing coping skills [59]. The remaining 4 miscellaneous apps were described as “wellness hacks” by Agapie et al [66]: Covid Coach, Daylio, Moodflow, and Talk Life. The full list of MHAs categorized by therapeutic approach is shown in Textbox 1.

**Therapeutic approach of the 20 mental health apps included in this review.**

**Cognitive behavioral therapy–based apps**

- Sanadak [65]
- Internet-based problem-solving therapy [56]
- ¡Aptívate! [54]
- iBobbly [57]
- Mindshift [66]
- Sanvello [66]
- Woebot [66]
- Wysa [66]

**Mindfulness-based apps**

- Headspace [66]
- Insight Timer [66]
- Shine [66]
- Smiling Mind [66]
- Mindful You [67]

**Miscellaneous apps**

- Prototype by McCall et al [58]
- Build Your Own Theme Song [63]
- BRAVE [59]
- Covid Coach [66]
- Daylio [66]
- Moodflow [66]
- Talk Life [66]

### Consideration of Racial and Ethnic Minority Groups While Developing MHAs

Of all the MHAs mentioned, 7 apps targeted racial and ethnic minority groups specifically: ¡Aptívate!, iBobbly, BRAVE, BYOTS, the prototype app by McCall et al [58], Sanadak, and Mindful You. The inclusion of racial and ethnic minority groups was ensured by codeveloping the app with the target population, by using workshops [56], interviews [55,59], usability trials [58], or working with culturally informed organizations [63]. ¡Aptívate! [54] was developed in Spanish language to be acceptable to the Hispanic population. Both Sanadak and Mindful You were developed with the specific needs of racial and ethnic minority groups in mind and tailored to the type of material used in the apps.

### Examining the Acceptability of MHAs Among Racial and Ethnic Minority Groups

To measure the acceptability of MHAs among racial and ethnic minority groups, 8 studies referred to app use and interactive data [54,56,57,59-61,65,67] in 6 MHAs. Studies reported good adherence to the specified MHAs throughout the set duration period in Hispanic, Aboriginal, Torres Strait Islander, and American Indian and Alaska Native individuals. Adherence data ranged from 81.8% to 91.2% of participants using and interacting with the app.

For the ¡Aptívate! app, participants were asked to use the app within the 8 weeks provided. The retention rate was 100% in the first week but decreased to 50% by the eighth week. This study suggests that the 50% drop in retention can be explained by the local versus remote recruitment of Latina participants. Those who attended baseline visits in person were more likely to use the app more frequently than those who did remotely. Dahne et al [54] also reported that 50% of Hispanic participants who continued using ¡Aptívate! 2 months after enrollment showed a high level of acceptability. Pratap et al [56] also recruited Hispanic and Latina participants and conducted a randomized controlled trial for 3 months to evaluate the iPST app. Engagement and retention rates were assessed based on the number of completed surveys. The study reported 34.4% dropouts in the Hispanic and Latino population. Of those who dropped out, more than half reported making ≤US $20,000 annually. Of those who used the app, Hispanic and Latina participants showed a 50% decrease in engagement from week 1 to week 4. It is important to note that this is based on the completion of the assessment and, therefore, is not an accurate representation of app use.

The BRAVE app was also used in an 8-week trial [60], with an overall retention rate of 87%. Among the participants in the BRAVE arm, 41 American Indian and Alaska Native participants opted out during the intervention and 25 opted out at crossover. This suggests a dropout rate of only 13% [59].

The iBobbly app was used by Aboriginal and Torres Strait Islander participants in a 6-week trial. The app had the lowest dropout rate (3%) of all other MHAs in this review. The study argues that this was due to technical issues and speculated that some participants might have felt self-conscious about sharing their use data [57].

Sanadak was designed for Syrian participants, and they were asked to use the app regularly for 4 weeks. The retention rate was 87.2%, with a dropout rate of 12.8%, where most participants refused to continue. Upon further analysis, Röhr et al [65] claimed that there was no significant difference between participants who completed the study and those who did not. Finally, Watson-Singleton et al [67] explored the Mindful You app. African American participants were asked to use the app for 2 weeks. The study reported a dropout rate of 45%, which the study found difficult to explain because the app was designed specifically for African Americans. Participants who continued using the app felt positive about Mindful You, giving the app 4.38 stars out of 5.

Overall, dropout rates were significantly higher among Hispanic and Latino participants than among non-Hispanic participants, with the latter staying on average 18.5 days longer.

Qualitative studies measured the acceptability of MHAs using interviews [55], workshops and focus groups [63], surveys [40,62], and questionnaires [58].

Participants were interviewed about the iBobbly app in terms of acceptability, cultural appropriateness, and whether the app provided help with their feelings and created distractions. The Aboriginal and Torres Strait Islander participants reported that iBobbly was acceptable, especially in terms of accessibility. Moreover, the participants felt a sense of privacy that was valued more than talking with a therapist or a family member. Participants also spoke of the “shame” attached to young Aboriginal people when asking for help, and so the iBobbly app was seen as culturally appropriate. Povey et al [64] also explored the iBobbly app and compared it with a therapist-led app. Aboriginal and Torres Strait Islander participants showed enthusiasm when they helped design the AIMhi- Y app [43]. Barnett et al [63] conducted focus groups regarding the BYOTS app designed for Black and biracial girls, and they were prompted to use the app 3 times a day for 1 week. This study did not mention dropout rates. However, using focus groups, the study showed that Black and biracial girls found the BYOTS app acceptable and useful in daily situations.

Agapie et al [66] explored a variety of apps with Hispanic and Latina participants. They were asked to use 1 app from each category weekly for a 5-week period and then complete a focus group at the end of each week. Every week participants would use a different app and then the last week they chose their favorite. There were no official use data; participants were asked to report how often they used the app, and they were most likely to report “a few days a week.” It seems that mindfulness apps were more acceptable, with 60% of participants reporting continued use. During the focus groups, participants noted that the apps were easy to use and accessible. However, the participants generally preferred to use the apps with more free content. Some participants reported concern about whether the app was validated by professionals and expressed the need to feel safe.

Both Lungu and Sun [40] and Maloney et al [62] used a questionnaire to assess the acceptability of MHAs in general. Of the 75.3% Asian American young adults who endorsed seeking mental health help on the web, only 22% were interested in MHAs [40]. Asian American participants were more likely to be in the “No therapy” and “Online only” groups compared with White participants. Similarly, 56% of the Jamaican participants were interested in using MHAs. However, shame, stigma, and embarrassment were reported to be the major barriers to seeking help. However, using a questionnaire, McCall et al [58] found that African American women reported that the prototype app was easy to use and provided culturally helpful information for anxiety and depression.

Overall, the apps were acceptable both quantitatively through use data and qualitatively, as participants described their engagement with and enthusiasm for the apps. However, dropout rates among some racial and ethnic minority groups remain high, and there is some discrepancy in the measurement of acceptability.

### Examining the Effectiveness of MHAs With Racial and Ethnic Minority Groups

#### Outcome Measures and Study Design

Nine of the 15 studies included in this review were quantitative [40,54,56,57,59,61,62,65,67], 4 used mixed methods [55,58,60,63], and 2 were qualitative [64,66].

In terms of quantitative studies, 3 studies [54,56,57] assessed depression levels in Hispanic and Indigenous Australian individuals. Two studies assessed mental health resilience in American Indian and Alaska Native populations [59,61]. One study assessed the levels of PTSD in Syrian refugees [65], and the other focused on mindfulness in Black African Americans [67]. The last 2 measured the receptiveness of web-based mental health support and MHAs with Jamaican [62] and Asian American [40] participants.

Four studies adopted mixed methods designs [55,58,60,63] and assessed Aboriginal and Torres Strait Islander, Black and biracial, and Indigenous Australian population. Three of these studies explored named MHAs: BRAVE [60], iBobbly [55], and BYOTS [63]. The remaining was an unnamed app, and the study explored its usability [58]. All studies, except for the one by Stephens et al [60], used surveys [55,58,62,63] and focus groups [63] or interviews [55,56] or cognitive walkthrough and think-aloud methods [58]. Stephens et al [60] did not use any measures, as they reported lessons learned from recruiting and engaging participants from the previous BRAVE study [59]. Outcome measures that were used by Tighe et al [55] and Povey et al [64] were appropriately translated to and validated in other languages to suit the ethnicity of the sample. Surveys, workshops, and interviews were developed and approved by mental health professionals of the target ethnicity.

Finally, the 2 qualitative studies focused on measuring the acceptability of the respective MHAs and discussing barriers to continued use [64,66]. All the outcome measures are presented in Table 1.

#### Effectiveness

Studies that explored a specific app assessed its effectiveness by using either weekly assessments [54,56,65] or pre- and postintervention changes in outcome measure scores [57,59,61,63,67]. Outcome measures were divided into clinical outcomes (eg, depression, anxiety, and suicidality) and other behavioral outcomes (eg, distress, resilience, and self-efficacy).

Quantitative studies on clinical outcomes that measured effectiveness using weekly assessments had inconclusive results. The ¡Aptívate! app [54] reported significantly lower depressive symptoms in Hispanic adults than in the no-treatment group; however, depressive symptoms did not differ on average across time between the 2 groups. Pratap et al [56] found improvement in depression scores among Hispanic and non-Hispanic participants, regardless of the treatment arm and ethnicity. However, they noted no evidence of any clinically meaningful changes between the iPST and the control group. The authors noted that only participants who reported severe depressive symptoms showed the greatest decline; however, this only lasted until week 4 of the study. Tighe et al [57] reported a decline in depressive symptoms among Aboriginal and Torres Strait Islander participants, but no significant reduction was observed in the primary outcome of suicidality in Indigenous Australian participants. In addition, no significant relationship between use time and any of the outcome measures was observed. The Sanadak app [65] also showed no significant differences in PTSD symptoms between the intervention and control groups after 4 weeks and 4 months of follow-up.

Other behavioral outcomes were also explored by quantitative studies. For instance, Pratap et al [56] explored functional impairment in addition to depressive symptoms and found no difference in disability outcomes across treatment arms and no difference between Hispanic and non-Hispanic participants. Tighe et al [57] explored psychological distress and impulsivity as secondary outcomes. The iBobbly app was associated with a significant decrease in Kessler Psychological Distress Scale scores after 6 weeks; however, there was no significant change in impulsivity [57]. Rushing et al [59] included the following secondary outcomes: self-efficacy, self-esteem, resilience, coping strategies, substance use, and cultural identity. They found that American Indian and Alaska Native participants who reported better health on average at baseline were more likely to report stronger cultural identity, cultural resilience, and positive coping strategies. No significant differences emerged in any of the primary outcomes of the BRAVE app (help seeking, self-efficacy related to mental health, and negative coping) [59,61]. A surprising finding for the BRAVE app was that higher scores on help-seeking attitude at baseline were associated with a decrease in the number of clicks or engagement with the app. However, it is important to note that Wrobel et al [61] reported that the engagement data were highly skewed, with some participants clicking an average of 3.4 times, but some users clicked 49 times. Finally, Röhr et al [65] included the secondary outcomes: self-efficacy, self-stigma, and resilience. They found that after using the Sanadak app, Syrian refugees showed no differences in any of the secondary outcomes, except for self-stigma. Syrian refugees reported lower levels of self-stigma following the use of the Sanadak app.

Quantitative evidence from mixed methods studies also showed inconclusive results on both clinical and behavioral outcomes. Neal-Barnett et al [63] concluded that Black and biracial girls who used the BYOTS app reported significantly lower negative and anxious thoughts on day 7 than on day 1. Although this study showed a positive result, the app was used for only 1 week, so there is still uncertainty regarding whether these improvements would last. Watson-Singleton et al [67] reported that Black African American participants who used Mindful You showed a significant decrease in stress levels after 2 weeks. They also showed increased capacity for emotional regulation and a significant increase in self-efficacy and mindfulness behaviors. However, there were no significant differences in the endorsements of mindfulness attributes, attitudes, or knowledge.

Finally, of the 2 qualitative studies, the one by Agapie et al [66] used a focus group to ask about the perceived effectiveness of the different apps that the participants used. Hispanic and Latina participants reported that all the apps used had small positive impacts on their mental health. Miscellaneous apps were ranked as the most effective in improving mental health well-being, followed by CBT apps and mindfulness apps. Povey et al [64] focused only on acceptability, whereas McCall et al [58] explored usability rather than the effectiveness of the app.

### Barriers to MHA Use Within Racial and Ethnic Minority Groups

Several barriers to MHAs were reported by the studies, ranging from cost to cultural appropriateness. Four studies did not explicitly report any barriers; however, they did highlight that not all clients may respond to self-guided treatment [54,58,63,65].

One of the most common barriers to using MHAs was the lack of personal touch. Aboriginal and Torres Strait Islanders, who used the iBobbly app, reported the need for more cultural content that related to their community [64]. This barrier was also true for American Indian and Alaska Native participants who used the BRAVE app [59]. Rushing et al [59] reported that due to the lack of representation in the media, participants reacted positively to both study arms, as they both contained cultural content. Participants who used the iBobbly app reported that such apps were not given enough community awareness and were therefore less likely to be used [64]. Rushing et al [59] also found that those with higher help-seeking tendencies were less likely to use the BRAVE app, which they hypothesized was because they were more likely to have support from people around them. The need for a personal touch was common even across the multiple apps explored by Agapie et al [66], with Hispanic and Latina participants reporting that the content was not specific enough for them.

The second most common barrier was stigma. Islander participants who used the iBobbly app reported that others may not engage with the app due to stigma surrounding mental health [55]. Pratap et al [56] also noted similar concerns among Hispanic participants when using the iPST app. Jamaican participants have gone as far as to describe using MHAs as embarrassing, relating to the stigma attached to receiving mental health support [62].

The third barrier was the cost. This was not so common but was mentioned by both Islander and Hispanic participants [64,66]. Agapie et al [66] found that Hispanic participants were more likely to use apps that were richer in free content compared with those that required a subscription. Other barriers included the repetitiveness of the MHA that was described by Rushing et al [59] as “message fatigue,” as lack of engagement was evident after the 10th text sent by the BRAVE app. Furthermore, literacy and language barriers were brought up by Islander participants who argued that some people in their community may not be comfortable using English [64]. Finally, technical issues were also identified as barriers to using MHAs. For instance, Tighe et al [55] failed to gather use data for 21 out of the 61 participants due to internet connectivity issues, a technical problem with their device, or an uncharged battery. Stephens et al [60] also noted that some participants lost access to their mobile phones and were thus unable to interact with the content of the BRAVE app.

## Discussion

### Principal Findings

This scoping review aimed to (1) describe the purposes of using MHAs in racial and ethnic minority groups, (2) examine the acceptability of MHAs among those groups, (3) examine the effectiveness of MHAs with the groups, (4) identify the barriers to MHA use within the groups, and (5) identify the gaps in the literature. Overall, our research pooled findings from 15 publications and highlighted important findings regarding the evidence related to MHA use among the racial and ethnic minority groups. Overall, MHAs were used for different purposes such as improving depression, decreasing psychological distress, increasing cultural resilience, and promoting help-seeking behavior. Fundamentally, most MHAs targeting racial and ethnic minority groups are underpinned by CBT and focus on depressive symptoms. In terms of acceptability, MHAs appear to be of interest among racial and ethnic minority groups; however, there is limited and mixed evidence of their effectiveness. Barriers to use include intervention-specific characteristics (eg, repetitiveness of the tasks), user-specific characteristics (eg, stigma), and technology-specific characteristics (eg, internet connectivity). Finally, several gaps in the literature, namely, the participant pool, MHAs design, study design, and study location, were identified. Taken together, these findings need to be considered to deepen our knowledge of MHA use and experiences among racial and ethnic minority groups.

Regarding evidence based on the intended purpose of using MHAs in racial and ethnic minority groups, most of the apps included in our review focused on depression and psychological distress. Although depression is one of the most common mental health disorders with a high prevalence among young people [68], the fact that it is one of the main purposes of the use of MHAs in this population is relevant. For instance, people from racial and ethnic minority backgrounds experience increased challenges compared with the majority population including social inequities, discrimination, and disparities in living conditions and work environments that may increase the risk of developing depression and psychological distress [26-28]. Islander participants who used the iBobbly app noted the need of more cultural content that was specific for them to increase engagement with the MHA [64]. However, Watson-Singleton et al [67] reported a 45% dropout rate from Mindful You despite having created a culturally specific app for Black African American population. Thus, there seems to be uncertainty about what specific changes would keep racial and ethnic minority groups engaged in MHAs. Another common challenge experienced by racial and ethnic minority groups is the stigma against mental health, which can form a barrier to accessing mental health support [33-35]. Stigma was a common barrier to using MHAs across Islander, Hispanic, and Jamaican participants [56,62,64]. Only one app in this review addressed this issue and focused on improving help-seeking behaviors [59]. However, the app reported no significant improvement in help-seeking behavior. In contrast, Röhr et al [65] found that a secondary outcome of the Sanadak app was reduced self-stigma in Syrian refugees. Therefore, future studies should aim to improve help-seeking behaviors and reduce mental health stigma in people from racial and ethnic minority backgrounds.

This review noted two critical observations regarding acceptability: (1) the measurements used and (2) attitudes of racial and ethnic minority groups toward MHAs. We followed the definition by Sekhon et al [52] for measuring acceptability: the willingness to participate and the adherence to the MHA. In our review, 8 studies measured acceptability using use or interactive data [54,56,57,59-61,64,67], and 7 used qualitative methods such as interviews, workshops, and surveys [40,55,58,62-64,66]. How acceptability is measured in these studies is essential, as it can affect how an MHA is perceived. For instance, in our review, the iBobbly app was investigated using both use data [55] and qualitative methods [57]. The findings showed that iBobbly was not acceptable in terms of use data; however, qualitative evidence showed that Aboriginal and Torres Strait Islanders reported that the iBobbly app was acceptable and culturally appropriate, and it reduced stigma surrounding mental health issues. Our review further highlights the heterogeneity in the definition and measurement of acceptability, making it difficult to draw conclusions.

Second, there seems to be ambivalence around racial and ethnic minority groups in terms of the acceptability of MHAs. Hispanic and Latina participants showed a high willingness to use MHAs [54,56] but showed a lack of engagement and high dropout rates [54,56]. In contrast, Agapie et al [66] found that 60% of Hispanic participants used the mindfulness apps even after the trial. Similarly, this review shows how many among Black and African American participants find MHAs useful and acceptable [62,63,67]. However, in the study by Watson-Singleton et al [67], almost half of the Black American participants dropped out for no given reason. In the study by Maloney et al [62] Jamaican participants explained stigma and embarrassment as major barriers to use. Similarly, the BRAVE app was found to be helpful for American Indian and Alaska Native participants; however, upon closer examination of the interactive data, Wrobel et al [61] found that engagement was lower than expected. The study that used the iBobbly app showed that Aboriginal and Torres Strait Islander participants were highly willing to use MHAs [55]; however, there was still a lack of interest in MHAs among them [55]. The other 2 racial and ethnic minority groups included in this study were Syrian refugees and Asian American individuals, who both showed high interest in MHAs and high dropout rates [66] or would rather use Facebook [40].

Overall, despite the high willingness of racial and ethnic minority groups to use MHAs, evidence reports an overall mixed view of engagement. More research adopting appropriate and standardized methods for measuring acceptability should be considered in the future.

Of note, 2 user-specific factors are related to the acceptability of MHAs. First is the level of psychological distress among racial and ethnic minority groups. In our review, we observed that Indigenous Australians with higher levels of distress were more likely to use MHAs and adhere to them [57]. However, in qualitative studies, Indigenous Australians and Aboriginal and Torres Strait Islanders reported that in extreme distress, they might not benefit from MHAs and FTF therapy would be more appropriate [55,64].

The second factor is help-seeking behavior as shown in the BRAVE study. Stephens et al [60] and Wrobel et al [61] found that surprisingly, those who scored high on help-seeking behavior showed less engagement with the BRAVE app. It was suggested that these participants might already have had their own ways to deal with distress and, therefore, were less likely to use other methods such as the BRAVE app. This corroborates the findings of Lungu and Sun [40], who suggest that some people from ethnic minority groups prefer to seek other forms of support (eg, Facebook). Facebook is not a MHA; however, some participants were more comfortable to reveal information on Facebook than attend FTF therapy. It would be helpful to understand the ways in which participants adapted to seek help, as it seems to influence engagement.

Regarding the effectiveness of the apps, the review found only 2 quantitatively effective apps: BYOTS and Mindful You [63,67], which corroborates the findings from previous reviews that reported limited or mixed evidence of the effectiveness of MHAs [17,21,69]. Previous reviews revealed that most MHAs claim effectiveness; however, there is no scientific evidence supporting their claims. This highlights the dire need for regulations on MHAs that are available on app stores. The BYOTS and Mindful You apps were also trialed for only 1 and 2 weeks, respectively; therefore, we cannot confidently assume that they will be effective for longer periods [63]. However, the fact that these MHAs were effective in reducing negative and anxious thoughts in Black and African Americans is in line with previous literature that showed that when given access to treatment, Black Americans benefit and engage more from therapy than White Americans [70]. Qualitative data found that Aboriginal youth in the study by Tighe et al [55] reported enjoying the iBobbly app even if it did not improve their clinical symptoms. Similarly, the participants who used the BRAVE app showed a significant positive improvement, but it was not different from those who received science, technology, engineering, and mathematics (STEM) messages [59]. Moreover, Sanadak app, which was used by Röhr et al [65], did not significantly decrease PTSD symptoms; however, participants’ self-stigma toward mental health was notably reduced. Overall, little is known about how users interact with MHAs in clinically meaningful ways.

In terms of app-specific factors that affect effectiveness, evidence shows that users prefer using mobile apps in short bursts of time [19] highlighting that long-term use might result in repetition for app users. A possible solution might be to have users regularly engage with the app to improve its benefits [22]. Stephens et al [60] suggested creating a “pause” in the BRAVE messages so that users continue to be engaged and avoid “text fatigue.” Future studies should investigate the features that can encourage engagement among MHAs users. This review highlights the importance of co-design approaches and cultural adaptations. Ramos et al [71] noted that culturally inspired MHAs may be more appealing to racial and ethnic minority groups and can lead to increased intervention uptake. All the MHAs included in this study, except ¡Aptívate! [54] and iPST [56], were designed with the guidance of racial and ethnic minority groups. The inclusion of these groups in the process of creating the app prevents stereotyping and ensures the most culturally relevant factors to the user [71]. ¡Aptívate! [54] and iPST [56] only included accessible language as a culturally adaptive factor in apps. This is in line with the review by Ramos et al [71], who found that almost 58% of the MHAs included only 1 criterion, suggesting that the inclusion of culturally relevant criteria is far from the norm. Our review also showed that a common barrier was that there was not enough cultural content, even for apps specifically designed for racial and ethnic minority groups [64]. Future studies should consider the impact of cultural factors on the effectiveness of MHAs. Furthermore, future studies would benefit from exploring these factors from a qualitative perspective for more insights, as this review shows only 1 effective MHA despite including many culturally adaptive factors.

### Observations and Gaps in the Field With Suggestions for Future Research

Four important observations emerged around the potential gaps in the literature: participant pool, MHA design, study design, and location of the study.

#### Participant Pool

The participants recruited in the studies that we reviewed were primarily Hispanic and Latina or Black and biracial. Therefore, there is a need to recruit participants in MHA research from a wider racial and ethnic minority background. Another important observation in the review is that only 2 studies [54,64] included a greater number of unemployed than employed participants. The remaining studies included either employed participants or those who attended colleges or schools. Future research should also include low-income racial and ethnic minority populations to help find ways to effectively incorporate MHA technology as an accessible mental health support tool.

#### MHA Design

Regarding the MHA design, the apps in this review were primarily CBT based [54,64] or inspired by it using acceptance-based therapy [55,57], problem-solving therapy [56], cognitive musical restructuring [63], or a mixture of psychotherapy and CBT [58,59,66]. Therefore, more research is needed to explore different theoretical underpinnings to identify what works for whom, in what context, and among different cultures.

#### Study Design

Most of the included studies were quantitative, which arguably did not explain why the apps were ineffective. Strategies such as interviews or workshops might help better explore the barriers experienced by participants and help tailor targeted interventions. For instance, studies with mixed methods design offered valuable insights into the strengths and barriers of MHAs [55,58,60,63]. Although qualitative research takes time, future MHA research should consider qualitative research as the beneficial next step to progress in the field of MHAs for racial and ethnic minority populations.

#### Study Location

A total of 10 studies were conducted in the United States [40,54,56,58-61,63,66,67], 2 in Australia [57,64], 1 in Canada [55], 1 in the United Kingdom [62],1 in Germany [65]. Overall, more studies are needed globally to achieve generalizability of the findings and improve our understanding of MHA use among people of racial and ethnic minorities. More research is needed to explore whether MHA might be incorporated into existing services as a source of additional support to help overcome some of the existing barriers to service receipt among racial and ethnic minority groups.

### Limitations

This review benefited from independent screening by 2 researchers, and this minimized selection bias. Similarly, 2 reviewers were involved in quality appraisal, thereby reducing any bias in the assessments. However, this study has some limitations. Although our search terms were guided by previous systematic reviews including racial and ethnic minority groups, this is not an extensive list of all terminology related to racial and ethnic minority groups; therefore, the review was limited to the search terms used. Moreover, as the researchers involved could only read English, several studies that may have been relevant to this review were excluded. However, despite not imposing limitations on the country of origin and an extensive list of racial and ethnic minority group–related search terms, we were only able to include 15 studies; this demonstrates a dearth of evidence of MHAs among racial and ethnic minority groups, which highlights the need for further investigation.

### Conclusions

In this review, we aimed to explore the use of MHAs among racial and ethnic minority groups. This review synthesized data from 15 publications and reviewed 7 interventions. Although acceptability seems fairly consistent, more research is needed to support MHA effectiveness and overcome existing barriers. Overall, the literature on MHAs among racial and ethnic minority groups is still scarce, and there is still much left to understand. Future app developers should consider including racial and ethnic minority groups’ input in the development of MHAs as well as widening the scope of MHAs to focus on a range of disorders and use different theoretical approaches.

## Appendix

Multimedia Appendix 1 PRISMA (Preferred Reporting Items for Systematic Reviews and Meta-Analyses) checklist for scoping reviews.

Multimedia Appendix 2 Scoping review search terms.

## Abbreviations

BYOTS: Build Your Own Theme Song
CBT: cognitive behavioral therapy
FTF: face-to-face
iPST: internet-based problem-solving therapy
MHA: mental health app
PRISMA: Preferred Reporting Items for Systematic Reviews and Meta-Analyses
PRISMA-ScR: Preferred Reporting Items for Systematic Reviews and Meta-Analyses extension for Scoping Reviews
PTSD: posttraumatic stress disorder
STEM: science, technology, engineering, and mathematics
