# Supplementary material for: Self-Guided Mental Health Apps Targeting Racial and Ethnic Minority Groups: Scoping Review
Source: JMIR Ment Health. 2023 Dec 6;10:e48991. doi: 10.2196/48991 (PMC10733819; doi:10.2196/48991)
Supplement: Multimedia Appendix 2 [file mental_v10i1e48991_app2.docx]

# Self-guided Mental Health Apps Targeting Racial and Ethnic Minority Groups: A Scoping Review

## Supplementary Material

### Appendix A: Scoping review search terms

Search made on 25.08.21 in Medline (Ovid), EMBASE (Ovid), PsycInfo (Ovid), PsychArticles (Ovid), PsychExtra (Ovid), Web of Science

Search results by Database

| Database | N |
| --- | --- |
| Medline (Ovid) | 371 |
| EMBASE | 562 |
| PsychINFO | 329 |
| PsychArticles | 1516 |
| PsychExtra | 17 |
| Web of Science | 824 |
| Total | 3619 |
| Duplicates | 972 |
| Tot after de-duplication | 2647 |

**Medline (OVID)**

1. Mobile Applications/ or exp Telemedicine/ or Telemedicine.mp. or ehealth.mp. or Mobile App*.mp. or "Mental health app".mp. or "mobile-based".mp. or "mhealth".mp. or "mental health application".mp. or "computer assisted diagnosis".mp. or "computer assisted therapy".mp. or "telepsychiatry".mp. or "telepsychology".mp. or "telemental health".mp. or "online therapy".mp. or "mobile devices".mp. or "cellular phones".mp. or "websites".mp. or "computers".mp. or "computer assisted therapy".mp.

Hits: 172837

1. ((exp Ethnicity/ or Ethnic.mp.) and Racial Minorities/) or "ethnicity".mp. or "race".mp. or "racial minority".mp. or "ethnic minority".mp. or "minority".mp. or "African American".mp. or "American Indian".mp. or "African".mp. or "Egyptian".mp. or "Ethiopian".mp. or "Caribbean".mp. or "Jamaican".mp. or "Nigerian".mp. or "South African".mp. or "Korean".mp. or "Vietnamese".mp. or "Asian".mp. or "Chinese".mp. or "Hispanic".mp. or "Spanish".mp. or "Latino".mp. or "Latina".mp. or "Latinx".mp. or "Puerto Ricans".mp. or "Cubans".mp. or "Mexicans".mp. or "Brazilian".mp. or "Filipino".mp. or "Arabic".mp. or "Algerian".mp. or "Armenian".mp. or "Pakistanis".mp. or "Iranian".mp. or "Somalis".mp. or "Turkish".mp. or "Indian".mp. or "Indonesian".mp. or "Indigenous".mp. or "native".mp. or "Native American".mp. or "First Nations".mp. or "aboriginal".mp. or "Asian American".mp. or "Black Minority".mp. or "BAME".mp. or "BIPOC".mp.

Hits: 1595226

1. exp Mental Health/ or exp Mental Disorders/ or "mental health".mp. or "mental disorder*".mp. or "mental disease*".mp. or "anxiety".mp. or "depression".mp. or "suicide".mp. or "affective disorder*".mp. or "mood disorder*".mp. or "Psychosis".mp. or "psychotic disorder*".mp. or "Obsessive compulsive disorder*".mp. or Autis*.mp. or " panic disorder*".mp. or "phobias".mp. or phobia*.mp. or "post traumatic stress disorder*".mp. or "bipolar".mp. or "schizophrenia".mp. or "eating disorder*".mp. or "anorexia".mp. or "bulimia".mp. or "binge-eating".mp. or "personality disorder*".mp. or "dissociative identity disorder*".mp. or "Attention deficit disorder*".mp. or "ADHD".mp.

Hits: 2166638

1. (Adolescen* or Teen* or Youth or Young Adult or Minors or Juvenile or Youngster or young adult* or late adolescen* or older adolescen* or adulthood transition or early adult* or emerging adult* or transition to adult* or young adult* or late adolescen* or older adolescen*).mp.

Hits: 2921495

1. 1 and 2 and 3 and 4

Hits: **371**

**PsychArticles (Ovid)**

1. (Mobile Applications OR Telemedicine OR Telemedicine OR ehealth OR Mobile App* OR "Mental health app" or "mobile-based" or "mhealth" or "mental health application" or "computer assisted diagnosis" or "computer assisted therapy" or "telepsychiatry" or "telepsychology" or "telemental health" or "online therapy" or "mobile devices" or "cellular phones" or "websites" or "computers" or "computer assisted therapy").mp

Hits: 9342

1. (Ethnicity OR Ethnic and Racial Minorities OR "ethnicity" or "race" or "racial minority" or "ethnic minority" or "minority" or "African American" or "American Indian" or "African" or "Egyptian" or "Ethiopian" or "Caribbean" or "Jamaican" or "Nigerian" or "South African" or "Korean" or "Vietnamese" or "Asian" or "Chinese" or "Hispanic" or "Spanish" or "Latino" or "Latina" or "Latinx" or "Puerto Ricans" or "Cubans" or "Mexicans" or "Brazilian" or "Filipino" or "Arabic" or "Algerian" or "Armenian" or "Pakistanis" or "Iranian" or "Somalis" or "Turkish" or "Indian" or "Indonesian" or "Indigenous" or "native" or "Native American" or "First Nations" or "aboriginal" or "Asian American" or "Black Minority" or "BAME" or "BIPOC").mp

Hits: 67555

1. (Mental Health OR Mental Disorders OR "mental health" or "mental disorder*" or "mental disease*" or "anxiety" or "depression" or "suicide" or "affective disorder*" or "mood disorder*" or "Psychosis" or "psychotic disorder*" or "Obsessive compulsive disorder*" or Autis* or" panic disorder*" or "phobias" or phobia* or "post traumatic stress disorder*" or "bipolar" or "schizophrenia" or "eating disorder*" or "anorexia" or "bulimia" or "binge-eating" or "personality disorder*" or "dissociative identity disorder*" or "Attention deficit disorder*" or "ADHD").mp

Hits: 92936

1. (Adolescen* or Teen* or Youth or Young Adult or Minors or Juvenile or Youngster or young adult* or late adolescen* or older adolescen* or adulthood transition or early adult* or emerging adult* or transition to adult* or young adult* or late adolescen* or older adolescen* ).mp

**Hits:47777**

1. 1 and 2 and 3 and 4

Hits: **1516**

**PsychExtra (Ovid)**

1. Mobile Applications/ or exp Telemedicine/ or Telemedicine.mp. or ehealth.mp. or Mobile App*.mp. or "Mental health app".mp. or "mobile-based".mp. or "mhealth".mp. or "mental health application".mp. or "computer assisted diagnosis".mp. or "computer assisted therapy".mp. or "telepsychiatry".mp. or "telepsychology".mp. or "telemental health".mp. or "online therapy".mp. or "mobile devices".mp. or "cellular phones".mp. or "websites".mp. or "computers".mp. or "computer assisted therapy".mp.

Hits: 3767

1. ((exp Ethnicity/ or Ethnic.mp.) and Racial Minorities/) or "ethnicity".mp. or "race".mp. or "racial minority".mp. or "ethnic minority".mp. or "minority".mp. or "African American".mp. or "American Indian".mp. or "African".mp. or "Egyptian".mp. or "Ethiopian".mp. or "Caribbean".mp. or "Jamaican".mp. or "Nigerian".mp. or "South African".mp. or "Korean".mp. or "Vietnamese".mp. or "Asian".mp. or "Chinese".mp. or "Hispanic".mp. or "Spanish".mp. or "Latino".mp. or "Latina".mp. or "Latinx".mp. or "Puerto Ricans".mp. or "Cubans".mp. or "Mexicans".mp. or "Brazilian".mp. or "Filipino".mp. or "Arabic".mp. or "Algerian".mp. or "Armenian".mp. or "Pakistanis".mp. or "Iranian".mp. or "Somalis".mp. or "Turkish".mp. or "Indian".mp. or "Indonesian".mp. or "Indigenous".mp. or "native".mp. or "Native American".mp. or "First Nations".mp. or "aboriginal".mp. or "Asian American".mp. or "Black Minority".mp. or "BAME".mp. or "BIPOC".mp.

Hits: 26454

1. exp Mental Health/ or exp Mental Disorders/ or "mental health".mp. or "mental disorder*".mp. or "mental disease*".mp. or "anxiety".mp. or "depression".mp. or "suicide".mp. or "affective disorder*".mp. or "mood disorder*".mp. or "Psychosis".mp. or "psychotic disorder*".mp. or "Obsessive compulsive disorder*".mp. or Autis*.mp. or " panic disorder*".mp. or "phobias".mp. or phobia*.mp. or "post traumatic stress disorder*".mp. or "bipolar".mp. or "schizophrenia".mp. or "eating disorder*".mp. or "anorexia".mp. or "bulimia".mp. or "binge-eating".mp. or "personality disorder*".mp. or "dissociative identity disorder*".mp. or "Attention deficit disorder*".mp. or "ADHD".mp.

Hits: 66305

1. (Adolescen* or Teen* or Youth or Young Adult or Minors or Juvenile or Youngster or young adult* or late adolescen* or older adolescen* or adulthood transition or early adult* or emerging adult* or transition to adult* or young adult* or late adolescen* or older adolescen*).mp.

Hits: 25084

1. 1 and 2 and 3 and 4

Hits: **17**

**PsychINFO (Ovid)**

1. Mobile Applications/ or exp Telemedicine/ or Telemedicine.mp. or Electronic Health Services.mp. or Mobile App*.mp. or "Mental health app".mp. or "mobile-based".mp. or "mhealth".mp. or "mental health application".mp. or "computer assisted diagnosis".mp. or "computer assisted therapy".mp. or "telepsychiatry".mp. or "telepsychology".mp. or "telemental health".mp. or "online therapy".mp. or "mobile devices".mp. or "cellular phones".mp. or "websites".mp. or "computers".mp. or "computer assisted therapy".mp.

Hits: 64438

1. ((exp Ethnic Identity/ or Ethnic.mp.) and Racial Minorities/) or "ethnicity".mp. or "race".mp. or "racial minority".mp. or "ethnic minority".mp. or "minority".mp. or "African American".mp. or "American Indian".mp. or "African".mp. or "Egyptian".mp. or "Ethiopian".mp. or "Caribbean".mp. or "Jamaican".mp. or "Nigerian".mp. or "South African".mp. or "Korean".mp. or "Vietnamese".mp. or "Asian".mp. or "Chinese".mp. or "Hispanic".mp. or "Spanish".mp. or "Latino".mp. or "Latina".mp. or "Latinx".mp. or "Puerto Ricans".mp. or "Cubans".mp. or "Mexicans".mp. or "Brazilian".mp. or "Filipino".mp. or "Arabic".mp. or "Algerian".mp. or "Armenian".mp. or "Pakistanis".mp. or "Iranian".mp. or "Somalis".mp. or "Turkish".mp. or "Indian".mp. or "Indonesian".mp. or "Indigenous".mp. or "native".mp. or "Native American".mp. or "First Nations".mp. or "aboriginal".mp. or "Asian American".mp. or "Black Minority".mp. or "BAME".mp. or "BIPOC".mp.

Hits: 482517

1. exp Mental Health/ or exp Mental Disorders/ or "mental health".mp. or "mental disorder*".mp. or "mental disease*".mp. or "anxiety".mp. or "depression".mp. or "suicide".mp. or "affective disorder*".mp. or "mood disorder*".mp. or "Psychosis".mp. or "psychotic disorder*".mp. or "Obsessive compulsive disorder*".mp. or Autis*.mp. or " panic disorder*".mp. or "phobias".mp. or phobia*.mp. or "post traumatic stress disorder*".mp. or "bipolar".mp. or "schizophrenia".mp. or "eating disorder*".mp. or "anorexia".mp. or "bulimia".mp. or "binge-eating".mp. or "personality disorder*".mp. or "dissociative identity disorder*".mp. or "Attention deficit disorder*".mp. or "ADHD".mp.

Hits: 1552302

1. (Adolescen* or Teen* or Youth or Young Adult or Minors or Juvenile or Youngster or young adult* or late adolescen* or older adolescen* or adulthood transition or early adult* or emerging adult* or transition to adult* or young adult* or late adolescen* or older adolescen*)

Hits: 712762

1. 1 and 2 and 3 and 4

Hits: **329**

**EMBASE (Ovid)**

1. mobile application/ or exp telemedicine/ or telemedicine.mp. or telehealth.mp. or mobile app*.mp. or "Mental health app".mp. or "mobile-based".mp. or "mhealth".mp. or "mental health application".mp. or "computer assisted diagnosis".mp. or "computer assisted therapy".mp. or "telepsychiatry".mp. or "telepsychology".mp. or "telemental health".mp. or "online therapy".mp. or "mobile devices".mp. or "cellular phones".mp. or "websites".mp. or "computers".mp. or "computer assisted therapy".mp.

Hits: 214720

1. ((exp ethnicity/ or Ethnic.mp.) and Racial Minorities/) or "ethnicity".mp. or "race".mp. or "racial minority".mp. or "ethnic minority".mp. or "minority".mp. or "African American".mp. or "American Indian".mp. or "African".mp. or "Egyptian".mp. or "Ethiopian".mp. or "Caribbean".mp. or "Jamaican".mp. or "Nigerian".mp. or "South African".mp. or "Korean".mp. or "Vietnamese".mp. or "Asian".mp. or "Chinese".mp. or "Hispanic".mp. or "Spanish".mp. or "Latino".mp. or "Latina".mp. or "Latinx".mp. or "Puerto Ricans".mp. or "Cubans".mp. or "Mexicans".mp. or "Brazilian".mp. or "Filipino".mp. or "Arabic".mp. or "Algerian".mp. or "Armenian".mp. or "Pakistanis".mp. or "Iranian".mp. or "Somalis".mp. or "Turkish".mp. or "Indian".mp. or "Indonesian".mp. or "Indigenous".mp. or "native".mp. or "Native American".mp. or "First Nations".mp. or "aboriginal".mp. or "Asian American".mp. or "Black Minority".mp. or "BAME".mp. or "BIPOC".mp.

Hits: 2071554

1. exp mental health/ or exp mental disease/ or "mental health".mp. or "mental disorder*".mp. or "mental disease*".mp. or "anxiety".mp. or "depression".mp. or "suicide".mp. or "affective disorder*".mp. or "mood disorder*".mp. or "Psychosis".mp. or "psychotic disorder*".mp. or "Obsessive compulsive disorder*".mp. or Autis*.mp. or " panic disorder*".mp. or "phobias".mp. or phobia*.mp. or "post traumatic stress disorder*".mp. or "bipolar".mp. or "schizophrenia".mp. or "eating disorder*".mp. or "anorexia".mp. or "bulimia".mp. or "binge-eating".mp. or "personality disorder*".mp. or "dissociative identity disorder*".mp. or "Attention deficit disorder*".mp.

Hits: 3306697

1. ("Adolescen*" or "Teen*" or "Youth" or "Young Adult" or "Minors" or "Juvenile" or "Youngster" or "young adult*" or "late adolescen*" or "older adolescen*" or "adulthood transition" or "early adult*" or "emerging adult*" or "transition to adult*" or "young adult*" or "late adolescen*" or "older adolescen*").mp.

Hits: 2439078

1. 1 and 2 and 3 and 4

Hits: **562**

**Web of Science (All datasets)**

(((TS=(("Mobile App*" or "Telemedicine" or "ehealth" or "Mental health app" or "mobile-based" or "mhealth" or "mental health app*" or "computer assisted diagnosis" or "computer assisted therapy" or "telepsychiatry" or "telepsychology" or "telemental health" or "online therapy" or "mobile devices" or "cellular phones" or "websites" or "computers" or "computer assisted therapy") )) AND TS=(("Ethnic*"or "Ethnic and Racial Minorities" or "ethnicity" or "race" or "racial minority" or "ethnic minority" or "minority" or "African American" or "American Indian" or "African" or "Egyptian" or "Ethiopian" or "Caribbean" or "Jamaican" or "Nigerian" or "South African" or "Korean" or "Vietnamese" or "Asian" or "Chinese" or "Hispanic" or "Spanish" or "Latino" or "Latina" or "Latinx" or "Puerto Ricans" or "Cubans" or "Mexicans" or "Brazilian" or "Filipino" or "Arabic" or "Algerian" or "Armenian" or "Pakistanis" or "Iranian" or "Somalis" or "Turkish" or "Indian" or "Indonesian" or "Indigenous" or "native" or "Native American" or "First Nations" or "aboriginal" or "Asian American" or "Black Minority" or "BAME" or "BIPOC") )) AND TS=(("mental health" or "mental disorder*" or "mental disease*" or "anxiety" or "depression" or "suicide" or "affective disorder*" or "mood disorder*" or "Psychosis" or "psychotic disorder*" or "Obsessive compulsive disorder*" or Autis* or" panic disorder*" or "phobias" or phobia* or "post traumatic stress disorder*" or "bipolar" or "schizophrenia" or "eating disorder*" or "anorexia" or "bulimia" or "binge-eating" or "personality disorder*" or "dissociative identity disorder*" or "Attention deficit disorder*" or "ADHD" ) )) AND TS=((Adolescen* or Teen* or Youth or Young Adult or Minors or Juvenile or Youngster or young adult* or late adolescen* or older adolescen* or adulthood transition or early adult* or emerging adult* or transition to adult* or young adult* or late adolescen* or older adolescen*))

Hits: **824**
